# Comparative Studies on Duplicated *foxl2* Paralogs in Spotted Knifejaw *Oplegnathus punctatus* Show Functional Diversification

**DOI:** 10.3390/genes14101847

**Published:** 2023-09-23

**Authors:** Xinxin Du, Haiyang Yu, Yujue Wang, Jinxiang Liu, Quanqi Zhang

**Affiliations:** 1 School of Life Science and Bioengineering, Jining University, Jining 273155, China; xinxindu@jnxy.edu.cn (X.D.); yuhaiyang@jnxy.edu.cn (H.Y.); 2 Key Laboratory of Marine Genetics and Breeding, Ministry of Education, College of Marine Life Sciences, Ocean University of China, No. 5 Yushan Road, Qingdao 266003, China; wyj@ouc.edu.cn (Y.W.); liujinxiang@ouc.edu.cn (J.L.)

**Keywords:** spotted knifejaw, whole genome duplication, *foxl2/2l*, molecular evolution, functional diversification

## Abstract

As a member of the forkhead box L gene family, *foxl2* plays a significant role in gonadal development and the regulation of reproduction. During the evolution of deuterostome, whole genome duplication (WGD)-enriched lineage diversifications and regulation mechanisms occurs. However, only limited research exists on *foxl2* duplication in teleost or other vertebrate species. In this study, two *foxl2* paralogs, *foxl2* and *foxl2l*, were identified in the transcriptome of spotted knifejaw (*Oplegnathus punctatus*), which had varying expressions in the gonads. The *foxl2* was expressed higher in the ovary, while *foxl2l* was expressed higher in the testis. Phylogenetic reconstruction, synteny analysis, and the molecular evolution test confirmed that *foxl2* and *foxl2l* likely originated from the first two WGD. The expression patterns test using qRT-PCR and ISH as well as motif scan analysis revealed evidence of potentially functional divergence between the *foxl2* and *foxl2l* paralogs in spotted knifejaw. Our results indicate that *foxl2* and *foxl2l* may originate from the first two WGD, be active in transcription, and have undergone functional divergence. These results shed new light on the evolutionary trajectories of *foxl2* and *foxl2l* and highlights the need for further detailed functional analysis of these two duplicated paralogs.

## 1. Introduction

As an evolutionarily conserved gene family, FOX gene was first identified in Drosophila melanogaster and named as *fkh* on account of the spiked head appearance caused by the mutations of this gene in adult fruit fly [1]. From yeast to human, a varied number of these gene family members were identified and divided into 19 subfamilies. The number of FOX genes varied among species. Four FOX genes were identified in yeast, 16 in fruit fly, 54 in puffer fish, and 50 in human [2,3,4]. Although all FOX gene family members have the FH domain, distinct roles have been occupied during evolution history [5]. Coupled with various signaling pathways including the TGFβ, MAPK, AKT/PKB, Hedgehog, and Wnt pathways, FOX genes participate in various biological processes, such as cell proliferation, immunoregulation, embryonic development, and organ differentiation [6,7,8,9].

As a member of the FOXL gene subfamily, *foxl2* plays important roles in sex differentiation and gametogenesis [10]. It has been found that *foxl2* represses male cues such as *sox9* in the ovary by regulating estrogen signaling and prevents the trans-differentiation of granulosa cells into Sertoli-like cells and theca cells into Leydig-like cells. It regulates steroidogenesis during gonad development as a repressor of steroidogenic enzymes such as *StAR*, *cyp11a1*, and *cyp17a1* [11,12,13]. As a sex-determining gene (SDG), *foxl2* plays important roles in goat and bivalve mollusks [14,15]. The knockout of *foxl2* in female mouse leads to sterility [16]. In XX Nile Tilapia, the mutation of *foxl2* also results in female to male sex reversal [17]. In addition, it also shows that *foxl2* is a female-biased gene in chicken [18], turtle [19], and frog [20]. All the forementioned studies suggest that *foxl2* is a conserved female reproductive factor. Additional *foxl2* genes have been widely identified in many teleost species and were originally named *foxl3* or *foxl2b,* including the European sea bass (*Dicentrarchus labrax*), rice field eel (*Monopterus albus*), medaka (*Oryzias latipes*), and zebrafish (*Denio rerio*) [21,22,23,24]. In Atlantic salmon and European sea bass, the gene *foxl3* was predominantly expressed in the testis [21,25,26]. Meanwhile, the expression of *foxl3* was restricted to the germ cells within the gonads, not the somatic cells in medaka. Adult female medaka with disrupted *foxl3* were observed to produce functional sperm in an expanded germinal epithelium, revealing the significance of *foxl3* as a germ cell–intrinsic factor in influencing the sperm–egg fate decision [23]. Further studies also found that *rec8* and *fbox47* could serve as the downstream targets of *foxl3* to regulate germline sex determination [27].

The additional *foxl2* paralog is initially attributed to the teleost-specific whole genome duplication (WGD). With the findings of the additional *foxl2* duplicates outside of the teleost lineage, it has been suggested that these two paralogs are originated from an ancestral gene at the base of the vertebrate by the first two rounds of the WGD [21]. In the NCBI database, the names of these two paralogs were *foxl2* and *foxl2l*, which were different from previous studies. To avoid confusion and make it easier for readers to search the related data directly in NCBI, we chose the names, *foxl2* and *foxl2l*, according to NCBI in this study.

The WGD have increased the complexity and genome size of vertebrates. Duplicated genes not only promote diversification and evolutionary innovation [28,29], but also undergoes different selective pressures and evolves into three fates according to the duplication-degeneration-complementation (DDC) model, nonfunctionalization, subfunctionalization, and neofunctionalization [30]. FOX genes are likely prone to evolutionary constraints due to their diverse and significant functions. As a key regulator of ovarian development, it had been shown that *foxl2* was under significant purifying selection pressure [31]. This might indicate that functional diversification may have occurred between the *foxl2* and *foxl2l* paralogs.

In this study, two *foxl2* paralogs, *foxl2* and *foxl2l*, were isolated in the spotted knifejaw transcriptome. To confirm the origin and evolutionary destiny of the duplicated *foxl2* and *foxl2l* paralogs, we performed phylogenetic reconstruction, chromosomal synteny analyses, and positive selection pressure tests in vertebrates. The results of qRT-PCR, in situ hybridization, and the motif scan analyses indicated the potential function diversification of these two paralogs in spotted knifejaw. This study enhances our understanding of the WGD and functional diversification in evolution history. It also provides a basis for studying the evolutions and functions of duplicated *foxl2* paralogs.

## 2. Materials and Methods

### 2.1. Fish and Sample Collection

Six healthy two-year-old spotted knifejaws, consisting of three females and three males, were randomly selected from Laizhou MingBo Aquatic Co., Ltd., Yantai, China and temporarily cultured in an institute aquarium tank at 25 °C with continuous aeration. The individuals were anesthetized with MS-222 and euthanatize by severing the spinal cord. The specimens, including the brain, kidney, heart, spleen, liver, ovary, and testis, were collected and snap-frozen in liquid nitrogen, then stored at −80 °C for subsequent RNA extraction.

### 2.2. Identification of FOXL Family Genes in Spotted Knifejaw

The FOXL gene family members of Nile tilapia (*Oreochromis niloticus*), medaka (*O. latipes*), and puffer fish (*Takifugu rubripes*) were retrieved from the NCBI online database and used as queries. To identify the FOXL gene family members of spotted knifejaw, a local BLAST with an E-value of 1 × 10^−5^ against the spotted knifejaw gonad transcriptome was used [32]. The accession numbers of all gene sequences utilized in this study are shown in Table S1.

### 2.3. Phylogenetic Analysis of FOXL Genes

To investigate the phylogenetic relationships and evolution fates of vertebrate FOXL genes, a phylogenetic reconstruction was performed using the maximum likelihood method. The Clustal X program with default parameters was employed to align the coding sequences of FOXL genes [33], and the resulting alignment file was provided in Supplementary File S1. JModelTest v2.1.4 was used to determine the appropriate substitution model and a maximum likelihood tree was constructed using phyML v3.1. The branch reliability was tested via bootstrap resampling with 1000 replicates. Species names and accession numbers used in phylogenetic reconstruction are shown in Table S1.

### 2.4. Synteny Analyses of Vertebrate foxl2 and foxl2l

The flanking genes of *foxl2* and *foxl2l* were performed to test the genes’ syntenic conservation. Online genome databases, including NCBI and Ensembl, were applied to find the flanking genes of *foxl2* and *foxl2l* in the genome of the selected species including *Haplochromis burtoni*, *Seriola dumerili*, *Perca flavescens*, *T. rubripes*, *O. niloticus*, *Lapisosteus oculatus*, *Callorhinchus milii*, *D. rerio*, *Geotrypetes seraphini*, *Chiroxiphia lanceolata*, *Homo sapiens*, *Xiphophorus maculatus*, *O. latipes*, and *Pelodiscus sinensis*. The genes were located and organized based on their relative positions within the chromosomes or scaffolds.

### 2.5. Protein Sequence Alignment and Motif Scan Analyses

The deduced *foxl2* and *foxl2l* amino acid sequences of the six selected species (*O. niloticus*, *O. punctatus*, *O. latipes*, *Gasterosteus aculeatus*, *Oncorhynchus mykiss*, and *Gadus morhua*) were aligned via Clustal X to test the similarities and differences between these two duplicates.

MEME suite was used to distinguish the possible functional divergence between *foxl2* and *foxl2l* [34]. The *foxl2* and *foxl2l* sequences of the selected species (*Astatotilapia burtoni*, *D. labrax*, *G. morhua*, *O. latipes*, *G. aculeatus*, *Metriaclima zebra*, *T. rubripes*, *O. niloticus*, *O. mykiss*, and *O. punctatus*) were used in this analysis.

### 2.6. RNA Extraction, cDNA Synthesis, and Tissue Distribution Analysis

In accordance with the manufacturer’s protocol, the TRIzol reagent (Invitrogen, Carlsbad, CA, USA) was used to extract total RNA from the specimens. To remove the genomic DNA and proteins, the DNase I (TaKaRa, Dalian, China) and RNAclean RNA kit (Biomed, Beijing, China) was applied. RNA quality and quantity were measured via agarose gel electrophoresis and the NanoPhotometer Pearl (Implen GmbH, Munich, Germany). Following the manufacturer’s instructions, the M-MLV kit (TaKaRa) was applied for cDNA synthesizing.

Specific primers for spotted knifejaw *foxl2* and *foxl2l* genes were designed via an online tool IDT (http://www.idtdna.com/Primerquest/Home/Index (accessed on 13 March 2023)) in 3′ untranslated regions (Table S2). A pre-experiment was performed to test the product specificity. Quantitative real-time PCR (qRT-PCR) was carried out using SYBR Premix Ex Taq II (TaKaRa) on Roche LightCycler 480 (Roche, Forrentrasse, Switzerland). The qRT-PCR data was analyzed via the 2^−∆∆Ct^ method and statistically processed using one-way ANOVA, where SPSS 20. *p* < 0.05 was considered as the statistical significance and all data were presented as the mean ± standard error of the mean (SEM).

Six 2-year-old spotted knifejaws were randomly selected (three females and three males) from a local fish farm for in situ hybridization (ISH). The testis and ovary samples were collected and fixed in 4% PFA overnight at 4 °C, dehydrated in gradients of increasing methanol, and then stored in 100% methanol at −20 °C. Then, the testis and ovary samples were embedded in paraffin after clearance via xylene. ISH was performed on the paraffin sections of the gonad specimens. ISH was performed using DIG-labeled RNA sense and antisense probes which were synthesized via the DIG RNA Labeling Kit (SP6/T7) (Roche, Mannheim, Germany) using the *foxl2/2l*-ISH-Fw/Rv-specific primers (Table S2).

### 2.7. Positive Selection Test for Duplicated foxl2 Genes

Eleven teleost species were selected to verify the differences in selective pressure between the *foxl2* and *foxl2l* genes. The screening criteria is according to the manual of PAML v4.7 [35]. Two Bayesian phylogenetic trees were constructed for the analysis. The CODEML package was used to estimate the selective pressures based on various site models such as M0, M1a, M2a, M3, M7, M8, and M8a. The ratio of nonsynonymous to synonymous (dN/dS) and the likelihood ratio test were used to confirm the positive selected sites. To detect changes in dN/dS, M0 and M3, nested pairs were compared. Additionally, the comparisons between M2a and M1a, M7 and M8, and M8a and M8 were applied to estimate the positively selected sites.

## 3. Results

### 3.1. Identification of Two foxl2 Paralogs in Spotted Knifejaw

Two *foxl2* paralogs, *foxl2* and *foxl2l*, were identified in the gonad transcriptome of spotted knifejaw via tblastx with an E-value of 1 × 10^−5^. According to the differential expression analysis results of the gonad transcriptome, *foxl2* was expressed higher in the ovary than the testis, while *foxl2l* was expressed higher in the testis than the ovary (Table S3).

### 3.2. Phylogeny Analysis of FOXL Genes

**Figure 1:**
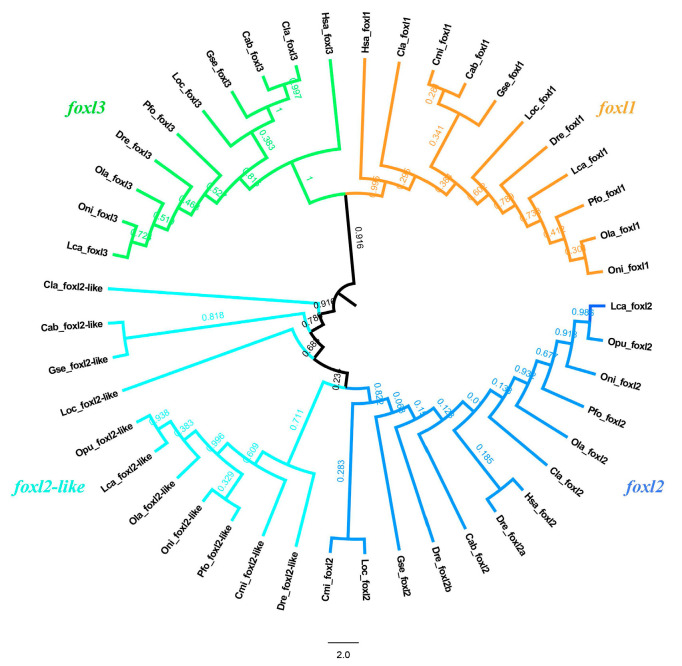
Phylogenetic analyses of FOXL gene family. Maximum likelihood method is applied to construct the phylogenetic tree. Numbers at the tree nodes are bootstrap values with 1000 replicates. Abbreviations: Oni, *O. niloticus*; Ola, *O. latipes*; Pfo, *Poecilia formosa*; Lca, *Lates calcarifer*; Dre, *D. rerio*; Loc, *L. oculatus*; Gse, *G. seraphini*; Cab, *Chelonoidis abingdonii*; Cmi, *C. milii*; Cla, *C. lanceolata*; Hsa, *H. sapiens*; Opu, *O. punctatus*.

To investigate the evolutionary trajectory of FOXL gene family members, FOXL genes were retrieved from various vertebrates, including fishes, amphibians, reptiles, aves, and mammals (Table S1). To verify the evolutionary relationships of FOXL gene family members among vertebrates, a maximum likelihood phylogenetic tree was constructed (Figure 1). FOXL genes were classified into four subfamilies, namely *foxl1*, *foxl2*, *foxl2l*, and *foxl3*. Both FOXL genes were conserved across all groups of vertebrates. *foxl2* and *foxl2l* clustered into one clade and *foxl1* and *foxl3* clustered into another clade, which indicated that not only *foxl2* and *foxl2l* but also *foxl1* and *foxl3* may also have originated from the WGD.

### 3.3. Synteny of foxl2 and foxl2l Paralogs

**Figure 2:**
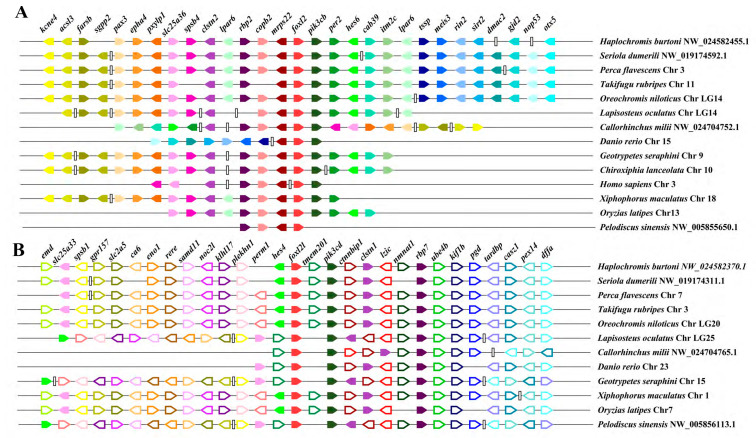
Chromosomal segments showing the synteny of *foxl2* and *foxl2l* paralogs. (**A**) Synteny analysis results of *foxl2*; (**B**) Synteny analysis results of *foxl2l*. Distinct genes are denoted via diverse colored pentagons, and their arrangement is established based on their relative location on the chromosome or scaffold. The gene names are indicated on top of each pentagon. The direction of the pentagon denotes the gene’s direction. Noncontiguous regions on the chromosome or scaffold are represented via vertical lines.

Synteny analysis was applied to test whether these two paralogs were originated from single gene duplication or the WGD. As shown in Figure 2, the two paralogs and their adjacent genes were positioned based on their relative locations and orientations on the scaffold or chromosome. The genes near *foxl2* were highly conserved and shared the same directions not in the teleost lineage but also in other vertebrate species, except for an inversion of *slc25a36* and *spsb4* in the upstream of *foxl2* in the human genome. Meanwhile, a long fragment consisting of several genes and some single genes were lost around *foxl2* of some species (Figure 2A). The genes flanking *foxl2l* were conserved and largely shared the same orientation among the teleost. A long fragment inversion consisting of the *slac25a33*, *spsb1*, *gpr157*, *slc2a5*, *ca6*, *eno1*, *rere*, *samd11*, *noc2l*, *klhl17*, *plekhn1*, *perm1*, and *hes4* genes was located in the upstream of *foxl2l* in the turtle, caecilian, and spotted gar genomes. *tmem201* was located in the downstream of *foxl2l* in most teleost, but in the upstream in spotted gar, turtle, elephant shark, zebrafish, and caecilian.

A genome-wide search of the FOXL family genes found only two paralogs of *foxl2*, *foxl2* and *foxl2l*, in all the vertebrate animals analyzed in this study, except for the zebrafish which has three paralogs, *foxl2a*, *foxl2b,* and *foxl2l*. The comparison on the flanking genes of the scaffolds containing *foxl2* and *foxl2l* identified many other likely gene family paralogous members located around *foxl2* and *foxl2l*, respectively, such as *pik3cb* and *pik3cd* of the PIK3 (phosphoinositide-3-kinase, catalytic subunit) family, *rbp2* and *rbp7* of the RBP (retinol binding protein) family, *nmnat3* and *nmnat1* of the NMNAT (nicotinamide nucleotide adenylytransferase) family, *clstn2* and *clstn1* of the CLSTN (calsyntenin) family, and *hes6* and *hes4* of the HES (hairy and enhancer of split) family. These likely paralogous genes between the *foxl2*- and *foxl2l*- containing scaffolds might be originated from their corresponding ancestral gene during the first two WGD and simultaneously with the duplication of *foxl2* and *foxl2l*. Hence, it could be speculated that *foxl2* and *foxl2l* near these genes might also arise from the first two WGD and are differentiated in function.

### 3.4. Protein Sequences Alignment and Motifs in foxl2 and foxl2l

In order to detect the similarity between *foxl2* and *foxl2l*, multiple amino acid alignments of deduced protein sequences of these two paralogs were constructed with the selected teleost species (Nile tilapia, spotted knifejaw, medaka, stickleback, rainbow trout, and Atlantic cod). As shown in Figure S1, *foxl2* genes shared high identities among different species and *foxl2l* showed similar results. However, the comparison between *foxl2* and *foxl2l* genes revealed a clear difference in their protein sequences. Only the FH domain was conserved in both *foxl2* and *foxl2l*, whereas the C-terminal region differed significantly.

Motif scan analysis was applied to test whether there existed differences between these two paralogs (Figure S2). Ten motifs were predicted out via the MEME online tool and represented by different colors. According to the results, either *foxl2* or *foxl2l* was highly conserved among the selected species. But, similar to the results retrieved from the protein sequence alignment (Figure S1), only three motifs (motif 3, 4, and 6) were conserved between *foxl2* and *foxl2l*, which suggested that they remained as the same functions on DNA binding using the same motif but have undergone functional diversification. Compared to *foxl2*, *foxl2l* has a low conservation and a lot of variations between different species. It can be speculated that *foxl2l* may be a newly generated gene and has a fast evolution rate.

### 3.5. Tissue Distribution of Two foxl2 Paralogs in Spotted Knifejaw

**Figure 3:**
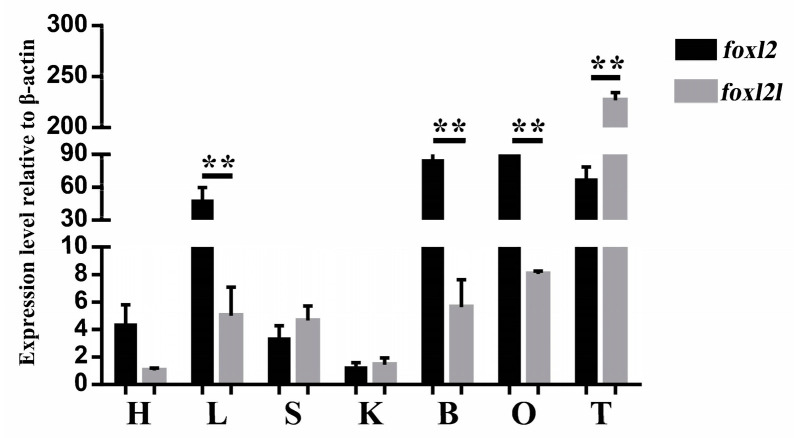
Expression patterns of *foxl2* and *foxl2l* in spotted knifejaw relative to *β-actin*. Data are shown as mean ± SEM (*n* = 3). The presence of asterisks denotes statistical significance, with a significance level of *p* < 0.05. Abbreviations: H, heart; L, liver; S, spleen; K, kidney; B, brain; O, ovary; T, testis.

qRT-PCR analysis was also applied to demonstrate the different organ-specific expression patterns of *foxl2* and *foxl2l* in spotted knifejaw (Figure 3). These two duplicated paralogs were expressed in all the organs selected in this analysis. The expression level of *foxl2* was significantly higher than that of *foxl2l* in somatic organs, including the liver and brain. Lower levels of *foxl2* were detected in the heart, spleen, and kidney, and no difference between the two paralogs was observed. The *foxl2* mRNA level was found to be higher in the ovary compared to *foxl2l*, while in the testis, a significantly higher expression of *foxl2l* was detected (Figure 3), which wis consistent with the transcriptome results.

**Figure 4:**
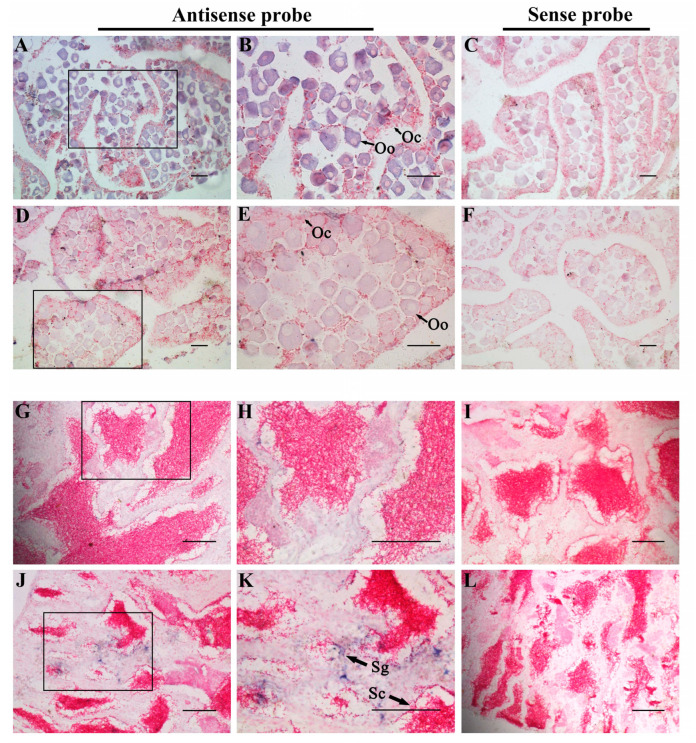
In situ hybridization of *foxl2* and *foxl2l* in spotted knifejaw gonad. The positive cells were stained purple or blue. Negative control with sense probe hybridization were unstained. (**A**) Antisense probe hybridization result of *foxl2* in ovary; (**B**) a local magnification of (**A**); (**C**) sense probe hybridization result of *foxl2* in ovary; (**D**) antisense probe hybridization result of *foxl2l* in ovary; (**E**) a local magnification of (**D**); (**F**) sense probe hybridization result of *foxl2l* in ovary; (**G**) antisense probe hybridization result of *foxl2* in testis; (**H**) a local magnification of (**G**); (**I**) sense probe hybridization result of *foxl2* in testis; (**J**) antisense probe hybridization result of *foxl2l* in testis; (**K**) a local magnification of (**J**); and (**L**) sense probe hybridization result of *foxl2l* in testis; Abbreviations: Oo, oogonia; Oc, oocytes; Sg, spermatogonia; Sc, spermatocytes. Scale bars = 50 μm.

In consideration of the different expression levels of *foxl2* and *foxl2l* in the spotted knifejaw gonad, the distribution of these two paralogs were detected in the gonads via ISH. The histological observation of the ovary revealed that it mainly consisted of oogonia and oocytes at stage I and stage II. Strong signals of *foxl2* mRNA were observed uniformly throughout the cytoplasm of both oogonia and oocytes (Figure 4A,B). Very light staining signals of *foxl2l* mRNA were tested in the ovary sections (Figure 4D,E). The testis sections mainly consisted of germ cells and Sertoli cells. No signals of *foxl2* were detected in the testis sections (Figure 4G,H), while signals of *foxl2l* were detected in spermatogonia and spermatocytes (Figure 4J,K). No signals were observed in the gonad sections via sense probes (Figure 4C,F,I,L). The expression profiles and tissue distributions can be clarified that functional divergence does have occurred between *foxl2* and *foxl2l*.

### 3.6. Molecular Evolution of Teleost foxl2 and foxl2l

During the evolution history, many SNP and random mutations were found in the protein sequences, which may potentially affect gene functions. With the purpose of testing the possibility of functional diversification, codon-based models (M0/M3, M1a/M2a, and M7/M8) in the PAML package were applied to examine the differences of selection pressure between *foxl2* and *foxl2l* (Table 1). The phylogenetic trees used for this analysis are shown in Figure S3. The comparison between M0 and M3 illustrated that both *foxl2* and *foxl2l* genes were under variable alternative pressures with site 41S in *foxl2l* being significantly selected under the pressures. Then, likelihood ratios were tested by comparing the pairs of M1a/M2a, M7/M8, and M8/M8a to the screen amino acid sites which were under positively selective pressures. After confirmation via the chi2 program in the PAML package, no site was positively selected in both the *foxl2* and *foxl2l* protein sequences, indicating that the *foxl2* and *foxl2l* genes were under purifying selection. In accordance with these, it can be speculated that there might be a diversification between *foxl2* and *foxl2l* during the evolution process.

## 4. Discussion

### 4.1. Expansion of Fox Family Genes

As an ancient class of DNA-binding transcription factors, FOX gene family members occupied various functions in cell proliferation, organ differentiation, embryonic development, and immunoregulation [6,7,8,9]. Forkhead proteins are widely involved in morphogenetic processes, suggesting that the increasing complexity in the genome and body plan may be the driving force behind the expansion of the FOX gene family [6]. Fox gene family members have been only found in opisthokont organisms, including animals and fungi. Typically, the higher the evolutionary status of this species, the more FOX genes can be found in the genome. The WGD not only leads to gene duplications, but also promotes the diversification of species and evolutionary innovation within the vertebrate genome [28,36,37]. After expanding via duplication, new duplicates typically evolve and acquire distinct functions [38,39]. According to the abundant vertebrate genome resources, we can still find evidences which support this speculation. Compared with mammals, many additionally duplicated FOX gene family paralogs originated from the teleost-specific WGD, which can be found in the teleost genomes such as two *foxb1*, two *foxc1*, two *foxf2,* and two *foxk2*. Overall, FOX gene family members have expanded via the WGD in evolution history. In this study, an expansion was also identified in the FOXL gene subfamily. Four FOXL genes, *foxl1*, *foxl2*, *foxl2l*, and *foxl3*, could be found in almost all vertebrate studied in this research.

### 4.2. foxl2 and foxl2l Are Generated from WGD at the Base of Vertebrate Radiation

Generally, new genes can emerge via a variety of evolutionary mechanisms, such as exon shuffling, retroposition, duplication, mobile elements, gene fission and fusion, lateral gene transfer, and de novo origination. Among these mechanisms, duplications consisted of a single gene duplication, segmental duplication, and the WGD plays significant roles in evolutionary innovation [40,41,42].

In this study, two *foxl2* paralogs, *foxl2* and *foxl2l*, were firstly identified and named in the spotted knifejaw genome. In vertebrates, transcription factors, ribosomal proteins, cyclins, and kinases are more frequently duplicated during the WGD process [43,44]. As shown in Figure 1, *foxl2* and *foxl2l* clustered into one clade, while *foxl1* and *foxl3* clustered into another clade, indicating that the duplicated transcription factor, *foxl2l*, may originated from the WGD, but not from the other evolutionary events. The additional paralogue *foxl2l* was initially thought to be resulted from the teleost-specific WGD, but the additional *foxl2l* was also found out of the teleost lineages such as amphibians, reptiles, aves, and mammals, indicating that *foxl2l* might have been duplicated from the ancestral *foxl2* gene earlier than 3R. The synteny analysis results also supported this speculation. As shown in Figure 2, the same genes conserved between the *foxl2* and *foxl2l* flanking fragments were not found. Many corresponding genes in the same gene family could be located around *foxl2* and *foxl2l*, such as *pik3cb* and *pik3cd*, *rbp2* and *rbp7*, *nmnat3* and *nmnat1*, and *clstn2* and *clstn1*. The results were consistent with the speculation that *foxl2* and *foxl2l* did not originate from the teleost-specific WGD but from the earlier first two WGD. According to the research and analysis of the correlation between *foxl2l* and *per3* in European sea bass, the same conclusion can be obtained [21].

### 4.3. Functional Diversification of Spotted Knifejaw foxl2 and foxl2l

After duplication, new gene pairs will undergo rapid changes in the sequences and structures, resulting in different evolution fates [45]. Generally, detrimental mutations will accumulate in one duplicate, where another copy maintains the initial functions [36]. In this study, it is hypothesized that *foxl2* and *foxl2l* had undergone functional diversification in vertebrates in accordance with the analyses in this study.

As shown in Figure S1, the FH domain was conserved between *foxl2* and *foxl2l*, which indicated primary functions in DNA binding. However, the amino acid sequences of these two paralogs were not conserved in the other parts, suggesting that functional diversification may have occurred between *foxl2* and *foxl2l*. Based on the comparation between M0 and M3, the *foxl2* and *foxl2l* paralogs were under selective pressure. However, no models of the positive selection were statistically significant for these two paralogs, suggesting that they likely underwent purifying selection during the course of evolution. Additionally, the MEME motif scan results showed that only three motifs (motif 3, motif 4, and motif 6) were conserved between these two paralogs while other parts of the protein sequences differed significantly, which verified the speculation that functional diversification has occurred between *foxl2* and *foxl2l*.

In the current study, the expression profiles of *foxl2* and *foxl2l* were performed via qRT-PCR. It showed that *foxl2* was expressed higher than *foxl2l* in the heart, liver, brain, and ovary, whereas it is contrary in the spleen, kidney, and testis. Meanwhile, the ISH results were consistent with the expression profiles, showing that *foxl2* was expressed higher than *foxl2l* in the ovary and lower in the testis. All results indicated a functional diversification between the *foxl2* and *foxl2l* paralogs. Previous studies have also shown different expression levels of *foxl2* and *foxl2l* in the gonads. In rainbow trout, *foxl2l* is expressed in the differentiating female gonad during the first oocyte meiosis but remains undetectable in males [46]. However, *foxl2l* genes of Atlantic salmon and European sea bass is predominantly expressed in the testis rather than the ovary in the adult stage, which is different from the profiles in rainbow trout. Even *foxl2l* is also expressed in the ovary, where the levels are much lower than *foxl2*. It can be indicated that *foxl2l* participates not only in the onset of oocyte meiosis, but also in regulating testis development [21,25]. In the half-smooth tongue sole, the expression profiles of *foxl2* and *foxl2l* are consistent with our results obtained from spotted knifejaw. It indicates that *foxl2* is mainly expressed in the ovary, while *foxl2l* is mainly expressed in the testis [47]. Sex differentiation and sex change are common phenomena during gonad development in teleost. The up-regulation of *foxl2* was detected in the gonads of sex-changed wrasse and half-smooth tongue sole [48,49]. In addition, *foxl2* was also highly up-regulated in the sex differentiation process of Chinese tongue sole [48]. All the results indicated a potential role of *foxl2* in sex change and sex differentiation. What function *foxl2l* plays in this process is unknown for the time being, and further studies are needed to investigate it.

Considering the aforementioned results, we speculate that *foxl2* and *foxl2l* shared conserved DNA-binding characteristics in the FH domain, whereas functional divergence had already occurred after their origination via the WGD. *foxl2* is predominantly expressed in the ovary and *foxl2l* in male germ cells. *Foxl2* coupled with *foxl2l* would have complementary roles in gonadal development and gametogenesis.

## 5. Conclusions

In this study, we investigated the origin and function of the *foxl2* and *foxl2l* paralogs in spotted knifejaw. We first characterized the *foxl2* and *foxl2l* paralogs in spotted knifejaw and speculated a potential origin via the first two WGD. Our findings suggest a probable functional diversification of these two duplicated paralogs in vertebrate evolution history. This study provided adequate information and new sights into the functional divergence of *foxl2* and *foxl2l* and can be treated as fundamental groundwork for following studies. Further analysis of the exact functions of *foxl2* and *foxl2l* remains to be addressed in suitable vertebrate species.

## Figures and Tables

**Table 1:** Results of sites’ model analyses on the *foxl2* and *foxl2l* gene tree.

Tree	Model	lnL	κ	Null	LRT	df	*p*-Value	Site	BEB
*foxl2*	M0	−3160.8085	2.55419	NA					
	M1a	−3154.2940	2.60048	NA					
	M2a	−3154.2940	2.60048	M1a	0	2	1		
	M3	−3134.7381	2.53049	M0	52.14	4	1.29 × 10^−10^		
	M7	−3134.8327	2.53409	NA					
	M8a	−3134.7717	2.53271	NA					
	M8	−3134.8345	2.53410	M7	0.004	2	0.998		
				M8a	0.13	1	0.718		
*foxl2l*	M0	−3681.4694	1.87394	NA					
	M1a	−3658.2278	1.95570	NA					
	M2a	−3658.2278	1.95570	M1a	0	2	1		
	M3	−3612.7257	1.93108	M0	137.5	4	0.0000	41S	0.954 *
	M7	−3615.4003	1.94152	NA					
	M8a	−3615.1257	1.94138	NA					
	M8	−3614.8491	1.94016	M7	1.1	2	0.577		
				M8a	0.55	1	0.458		

lnL: ln likelihood; κ: Transition/transversion ratio; df: Degrees of freedom; NA: not Applicable; *: Sites under positively selective pressure.

## Data Availability

The gene sequences used in this study and alignment file could be found in Supplementary files. Additional helps are available from the corresponding author upon reasonable request.

## Supplementary Materials

The following supporting information can be downloaded at: https://www.mdpi.com/article/10.3390/genes14101847/s1, Figure S1: Sequence alignment of the deduced *foxl2* and *foxl2l* protein sequences. Abbreviations: Oni, *O. niloticus*; Opu, *O. punctatus*; Ola, *O. latipes*; Gac, *G. aculeatus*; Omy, *O. mykiss*; Gmo, *G. morhua*. Figure S2: MEME motif scan results. Abbreviations: Abu, *A. burtoni*; Dla, *D. labrax*; Gmo, *G. morhua*; Gac, *G. aculeatus*; Mze, *M. zebra*; Omy, *O. mykiss*; Oni, *O. niloticus*; Ola, *O. latipes*; Tru, *T. rubripes*; Opu, *O. punctatus*. Figure S3: Phylogenetic tree of *foxl2* and *foxl2l* used in PAML analysis. Abbreviations: Abu, *A. burtoni*; Mze, *M. zebra*; Opu, *O. punctatus*; Dla, *D. labrax*; Ola, *O. latipes*; Omy, *O. mykiss*; Gmo, *G. morhua*; Tru, *T. rubripes*; Tni, *Tetraodon nigroviridis*; Gac, *G. aculeatus*; Oni, *O. niloticus*; Table S1: Species and Genes used in this study; Table S2: Primers used in this study; Table S3: The differential expression analysis results of the *foxl2* and *foxl2l*. Supplementary File S1: The alignment file of gene sequences used for phylogenetic analysis of FOXL genes.
