# Supplementary material for: Comparative Studies on Duplicated foxl2 Paralogs in Spotted Knifejaw Oplegnathus punctatus Show Functional Diversification
Source: Genes (Basel). 2023 Sep 23;14(10):1847. doi: 10.3390/genes14101847 (PMC10606028; doi:10.3390/genes14101847)
Supplement: Supplementary file 1 [file genes-14-01847-s001.zip › supplementary file/Figure S1.pdf]

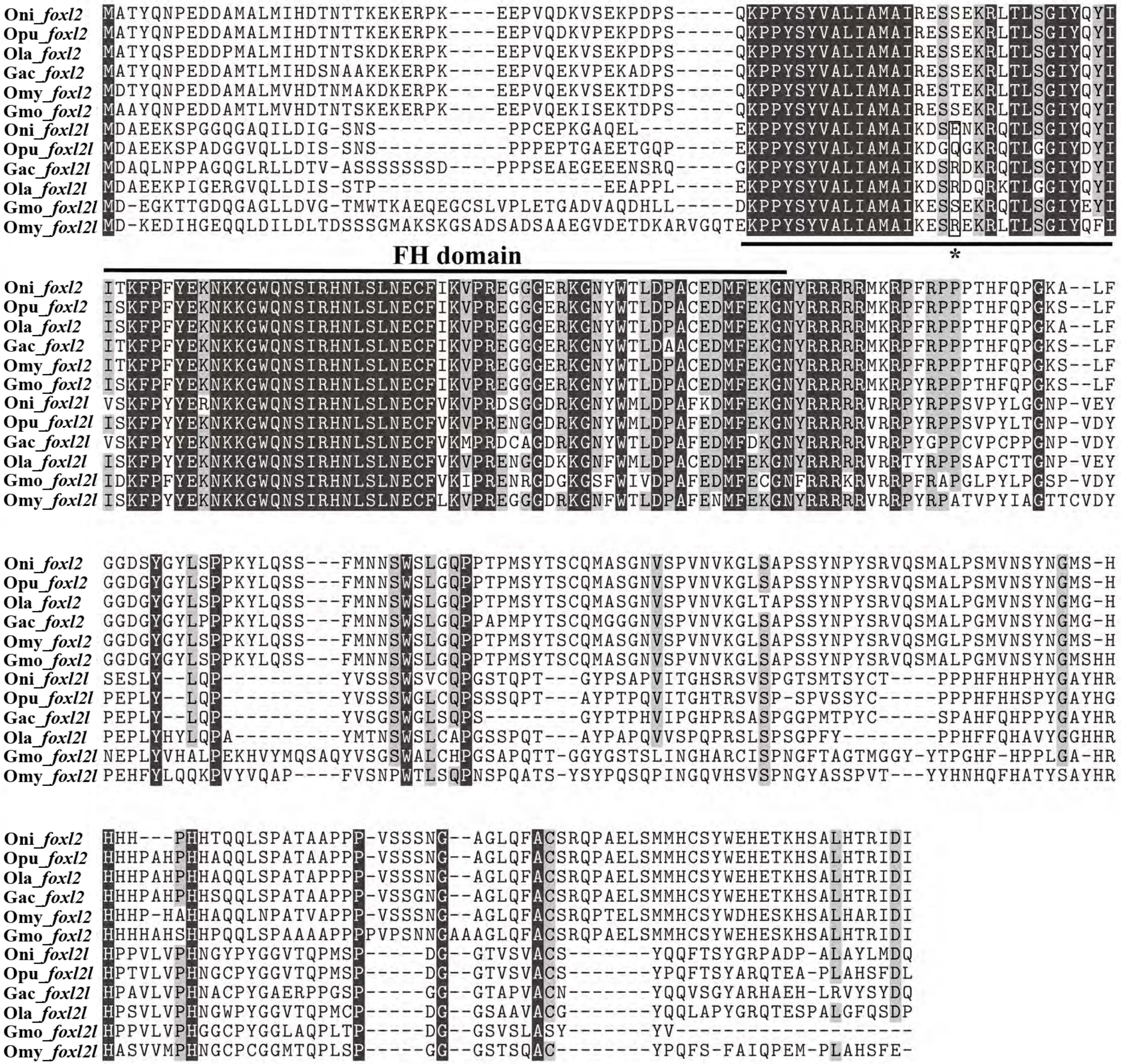

Oni_foxl2
Opu_foxl2
Ola_foxl2
Gac_foxl2
Omy_foxl2
Gmo_foxl2
Oni_foxl2l
Opu_foxl2l
Gac_foxl2l
Ola_foxl2l
Gmo_foxl2l
Omy_foxl2l
FH domain
*
