# Supplementary material for: Comparative Studies on Duplicated foxl2 Paralogs in Spotted Knifejaw Oplegnathus punctatus Show Functional Diversification
Source: Genes (Basel). 2023 Sep 23;14(10):1847. doi: 10.3390/genes14101847 (PMC10606028; doi:10.3390/genes14101847)
Supplement: Supplementary file 1 [file genes-14-01847-s001.zip › supplementary file/Table S1.pdf]

SPECIES	FOXL1	FOXL2	FOXL2l	FOXL3
Oplegnathus punctatus		OP123570.1	OP123571.1	
Lates calcarifer	XM_018704013.1	XM_018684117.1	XM_018665867.1	XM_018687806.1
Oreochromis niloticus	XM_005449217.4	NM_001279778.1	XM_005478201.4	XM_003438562.5
Maylandia zebra		XM_004562801.2	XM_004554201.1	
Poecilia formosa	XM_007567539.2	XM_007555965.2	XM_007577326.2	XM_007542632.2
Takifugu rubripes		XM_003968745.3	XM_003963226.3	
Tetraodon nigroviridis		ENSTNIT00000017555.1	ENSTNIT00000018443.1	
Gadus morhua		XM_030337297.1	XM_030358894.1	
Oryzias latipes	NM_001122919.2	NM_001104888.1	XM_004070665.4	XM_011487873.3
Lepisosteus oculatus	XM_015367956.1	XM_006637595.2	XM_015337030.1	XM_006637364.2
Danio rerio	NM_200984.1	XM_021481464.1	NM_001128810.1	
		NM_001317761.1		
Astatotilapia burtoni		NW_024582455.1	NW_024582370.1	
Dicentrarchus labrax		KF208536.1	JQ772483.2	
		ENSTNIG00000014321	ENSTNIG00000012897	
Oncorhynchus mykiss		XM_021581496.2	NM_001124484.1	

Gasterosteus aculeatus		XM_040191291.1	XM_040194533.1	
Callorhinchus milii	XM_007889238.2	XM_007888129.2	XM_007902179.2	
Geotrypetes seraphini	XM_033941309.1	XM_033959394.1	XM_033922419.1	XM_033962914.1
Chelonoidis abingdonii	XM_032793811.1	XM_032801425.1	XM_032775521.1	XM_032771021.1
Chiroxiphia lanceolata	XM_032701484.1	XM_032697658.1	XM_032703837.1	XM_032711676.1
Homo sapiens	NM_005250.3	NM_023067.4		NM_001374838.1
