# Supplementary material for: Comparative Studies on Duplicated foxl2 Paralogs in Spotted Knifejaw Oplegnathus punctatus Show Functional Diversification
Source: Genes (Basel). 2023 Sep 23;14(10):1847. doi: 10.3390/genes14101847 (PMC10606028; doi:10.3390/genes14101847)
Supplement: Supplementary file 1 [file genes-14-01847-s001.zip › supplementary file/Table S2.pdf]

Primers	Sequences
FOXL2-qpcr-FW	GAGAGCTGATTCCTGTGATTT
FOXL2-qpcr-RV	TGTGTCCTGATGACTTCTTTAC
FOXL2l-qpcr-FW	GACTCAAAGTTCATCTGTTTCAC
FOXL2l-qpcr-RV	AGTACCGACAATAATTCCTTGG
FOXL2-ISH-FW	ATTTAGGTGACACTATAGAAGGGGGGAACTACAG GAGACG
FOXL2-ISH-RV	TAATACGACTCACTATAGGGAGAGAGCAAGCGAA CTGAAG
FOXL2l-ISH-FW	ATTTAGGTGACACTATAGAAGGGTTCCACCACTCT CCTTAC
FOXL2l-ISH-RV	TAATACGACTCACTATAGGGAGAGAAACTGTAGCC TATCAGAAC
