# Supplementary material for: Comparative Studies on Duplicated foxl2 Paralogs in Spotted Knifejaw Oplegnathus punctatus Show Functional Diversification
Source: Genes (Basel). 2023 Sep 23;14(10):1847. doi: 10.3390/genes14101847 (PMC10606028; doi:10.3390/genes14101847)
Supplement: Supplementary file 1 [file genes-14-01847-s001.zip › supplementary file/Table S3.pdf]

Gene name	Basemean_testis (FPKM)	Basemean_ovary (FPKM)	Foldchange	Log2FC	P value
foxl2	3.68	138	37.47	5.23	3.02E-6
foxl2l	303.14	156.82	0.52	-0.95	0.38
