# Supplementary figures and images for: Comparative Studies on Duplicated foxl2 Paralogs in Spotted Knifejaw Oplegnathus punctatus Show Functional Diversification
Source: Genes (Basel). 2023 Sep 23;14(10):1847. doi: 10.3390/genes14101847 (PMC10606028; doi:10.3390/genes14101847)

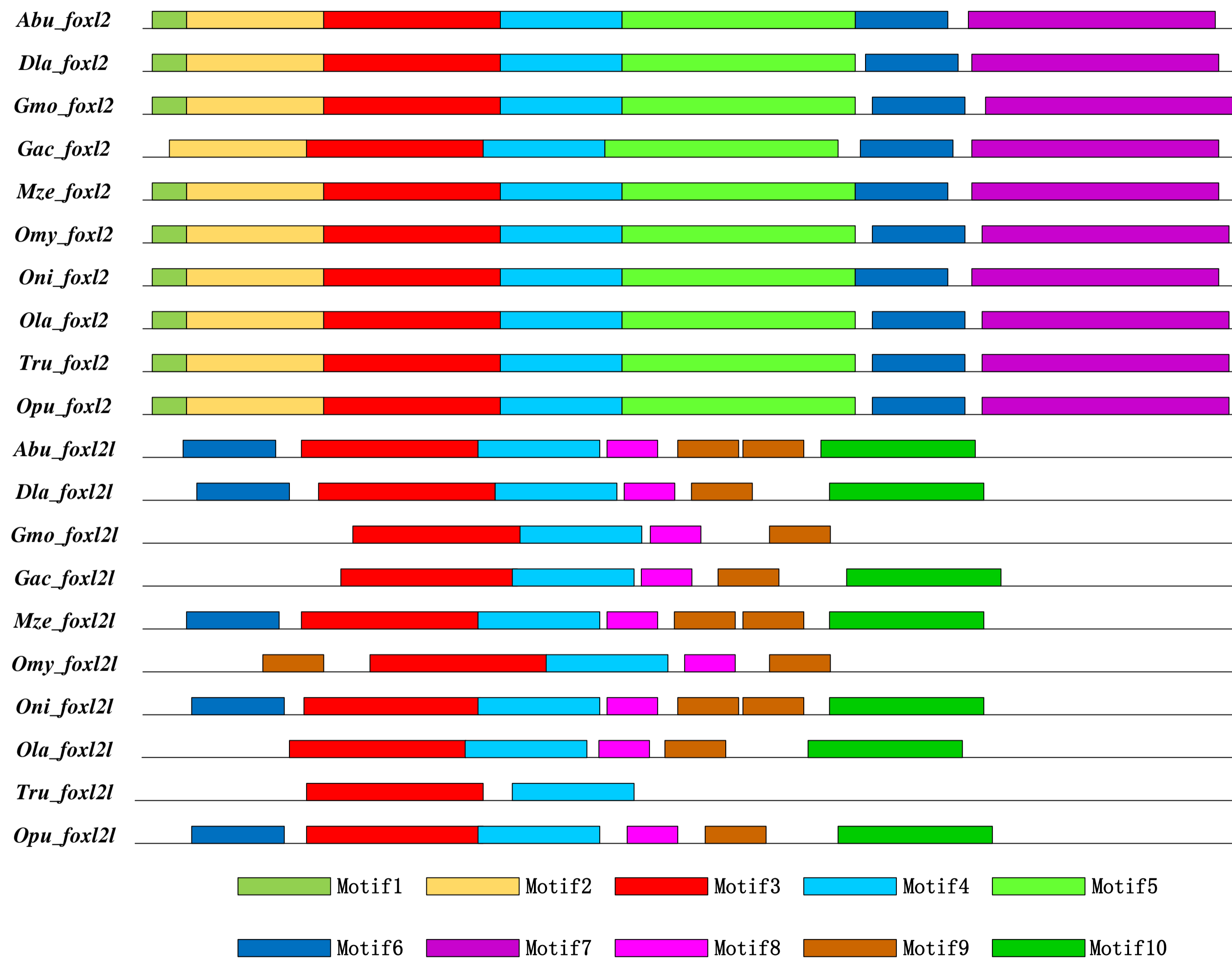

Supplement: Supplementary file 1 [file genes-14-01847-s001.zip › supplementary file/Figure S2.pdf]

A

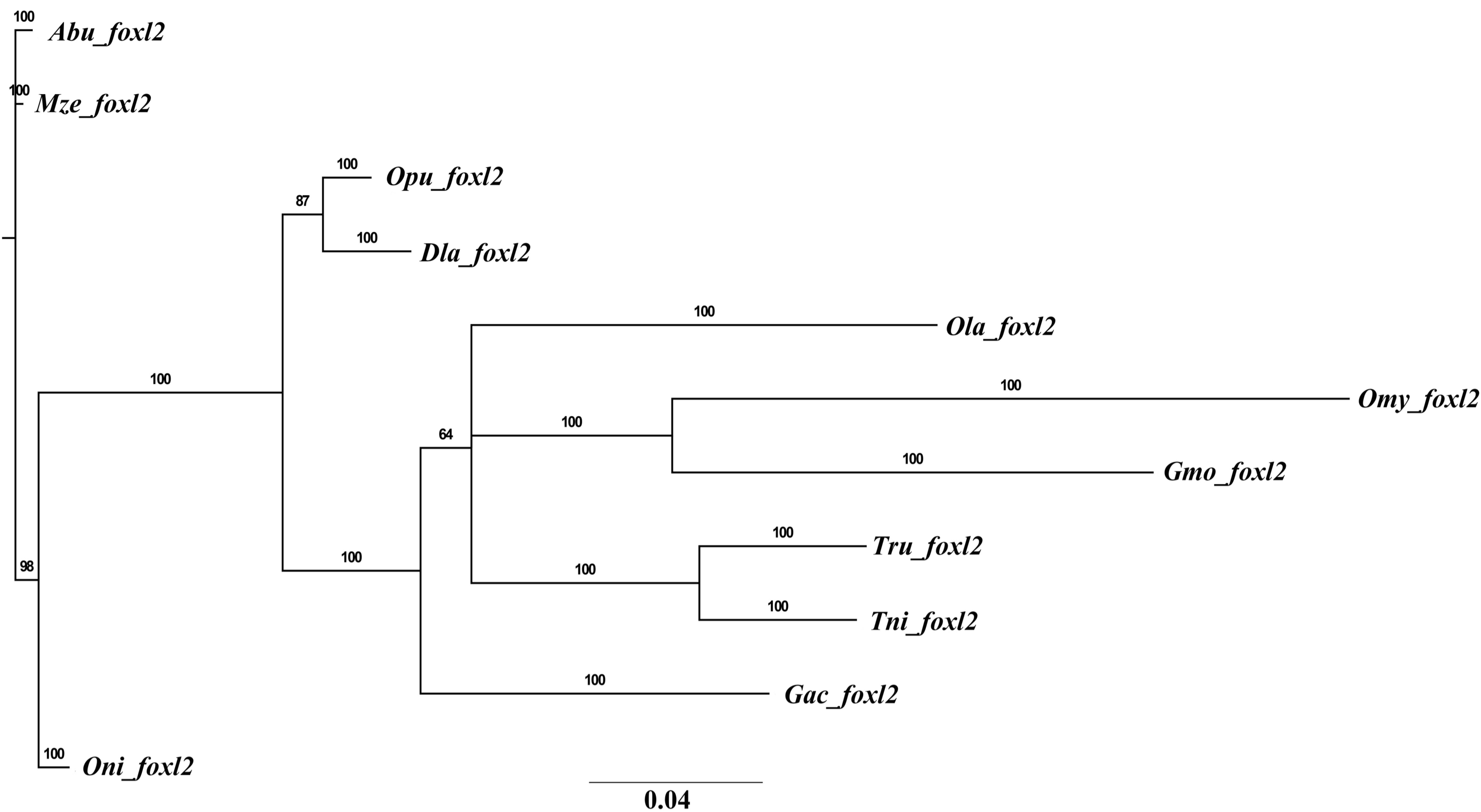

B

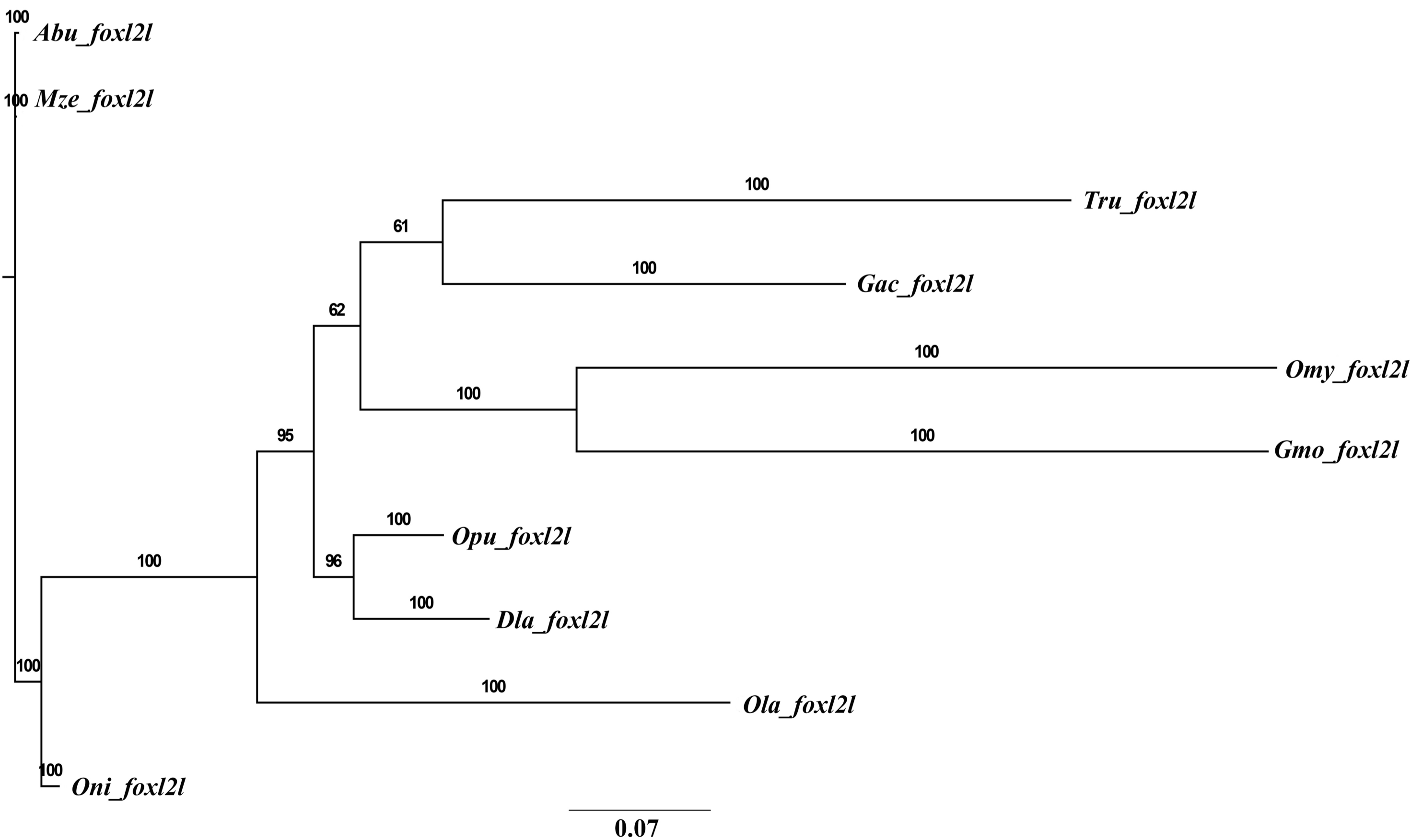

Supplement: Supplementary file 1 [file genes-14-01847-s001.zip › supplementary file/Figure S3.pdf]
